# Experimental Study on Fluorine Release from Photovoltaic Backsheet Materials Containing PVF and PVDF during Pyrolysis and Incineration in a Technical Lab-Scale Reactor at Various Temperatures

**DOI:** 10.3390/toxics7030047

**Published:** 2019-09-18

**Authors:** Philipp Danz, Venkat Aryan, Edda Möhle, Nicole Nowara

**Affiliations:** 1 Process engineering, Fraunhofer Institute for Environmental, Safety, and Energy Technology UMSICHT, 46047 Oberhausen, Germany; philipp.danz@umsicht.fraunhofer.de; 2 Sustainability and Resources Management, Fraunhofer Institute for Environmental, Safety, and Energy Technology UMSICHT, 46047 Oberhausen, Germany; 3 Analytics, Fraunhofer Institute for Environmental, Safety, and Energy Technology UMSICHT, 46047 Oberhausen, Germany; edda.moehle@umsicht.fraunhofer.de (E.M.); nicole.nowara@umsicht.fraunhofer.de (N.N.)

**Keywords:** pyrolysis and incineration, fluoropolymers, end-of-life treatment, PVDF/PVF, PV backsheet

## Abstract

With a sharp increase in photovoltaic (PV) installations across the world, PV waste is now a relatively new addition to the e-waste category. From 45,000 tonnes in 2016, the PV waste stream is rapidly increasing and is projected to reach 60 million tonnes by 2050. Backsheets are composite structures made from several material layers of polymer, adhesive, and primer. Widely used PV backsheets can be classified into three core types: (a) KPK (Kynar^®^/polyethylene terephthalate (PET)/Kynar^®^), (b) TPT (Tedlar^®^/PET/Tedlar^®^), and (c) PPE (PET/PET/ethylvinylacetate). Kynar^®^ and Tedlar^®^ are based on polyvinylidene fluoride (PVDF) and polyvinyl fluoride (PVF), respectively. PPE backsheets are fluorine-free composites made primarily from PET. With increasing focus on the end-of-life (EoL) handling of PV waste, the handling of fluoropolymers, which is largely unexplored, requires closer examination to avoid environmental damage. The aim of this study was to obtain information on the fluorine released from PV backsheet materials into the gas phase during combustion and pyrolysis as EoL pathways. Therefore, several experimental trials were conducted to measure fluorine transfer into the gas phase at 300 °C, 400 °C, 500 °C, and 900 °C (for pyrolysis) and at 750 °C, 850 °C, and 950 °C (for incineration).

## 1. Introduction

Electronic waste (e-waste) poses a twofold problem. On one hand, it is one of the most rapidly growing waste streams in the world, posing a significant threat to the environment [1,2,3]. On the other hand, due to its complex compositions, e-waste is also one of the most challenging waste streams to process [4,5,6]. With a sharp increase in photovoltaic (PV) installations across the world, PV waste is now a relatively new addition to the e-waste category [6,7]. From 45,000 tonnes in 2016, the PV waste stream is rapidly increasing and is projected to reach 60 million tonnes by 2050 [7,8].

Several studies have explored the recovery of valuables such as silver, copper, indium, gallium, tellurium, aluminum, etc. [9,10,11]; or the handling of hazardous metals such as cadmium, arsenic, lead, antimony, etc. [12,13]; or the treatment of typical polymers such as polyethylene terephthalate (PET), PE, PVC, etc. [14,15,16]. The handling of fluoropolymers, however, is largely unexplored. PV panels typically contain 0.4 kg backsheet/m^2^ panel or about 3 wt. % (weight percent) of backsheet material per PV panel [8]. This totals about 800,000 tonnes of PV backsheet waste that will have to be properly processed in light of the 75 GW PV capacity installed globally [17].

Backsheets are composite structures made from several material layers of polymer, adhesive, and primer. The widely used PV backsheets can be classified into three core types: (a) KPK (Kynar^®^/PET/Kynar^®^), (b) TPT (Tedlar^®^/PET/Tedlar^®^), and (c) PPE (PET/PET/ethylvinylacetate (EVA)). Kynar^®^ and Tedlar^®^ are both industrial names for fluoropolymers based on polyvinylidene fluoride (PVDF) and polyvinyl fluoride (PVF), respectively. PPE backsheets are fluorine-free composites made primarily from PET.

Fluoropolymers are hard to thermally degrade, can increase the potential formation of dioxins when burnt, and can pose health risks if material leaches into the atmosphere and contaminates soil and waterbodies [18,19,20,21]. The most widely used end-of-life (EoL) pathways for handling e-waste today are incineration and pyrolysis [16,22,23,24,25]. However, the thermal treatment of fluoropolymers is challenging due to their high thermal stability derived from the carbon–fluorine bond which is 490 kJ/mol, compared to the carbon–hydrogen bond at 420 kJ/mol or carbon–carbon bond at 340 kJ/mol [26]. As a result, fluoropolymers burn with a low heat release rate [22] and must be thermally treated at higher temperatures compared to typical engineering plastics [27].

Several literature studies thus far have focused on the incineration of the fluoropolymer type PTFE (polytetrafluoroethylene), owing to its wide commercial application as Teflon, and have observed the thermal degradation results on the formation of hydrocarbons, halogenated hydrocarbons, halogenated aromatics, carbonyl fluoride (COF_2_), and hydrogen fluoride (HF) [20,22,27]. Likewise, the pyrolysis of PTFE [18,28,29] was found to yield very high amounts of monomers. However, the pyrolysis of hydrofluorocarbon polymers such as PVF and PVDF was found to yield volatile fractions with high amounts of HF, which is detrimental to the environment and human health if handled improperly [18]. Furthermore, the pyrolysis oil produced also contains fluorinated hydrocarbons and thus poses a potential environmental hazard [17]. Hence, for establishing a technically feasible EoL treatment for PV backsheets, it is vital to study the fluorine release behavior from hydrofluorocarbon polymers such as PVDF and PVF during their thermal decomposition.

## 2. Materials and Methods

### 2.1. Experimental Setup and Methodology

The aim of this study was to identify whether and to what extent fluorine-based PV backsheets exhibit a fluorine release into the gas phase during their thermal decomposition. The experiments were carried out on three backsheet materials, namely, KPK (Kynar^®^/PET/Kynar^®^), TPT (Tedlar^®^/PET/Tedlar^®^), and PPE (PET/PET/EVA). Pyrolysis and incineration were the two thermal decomposition pathways analyzed in this study.

**Figure 1:**
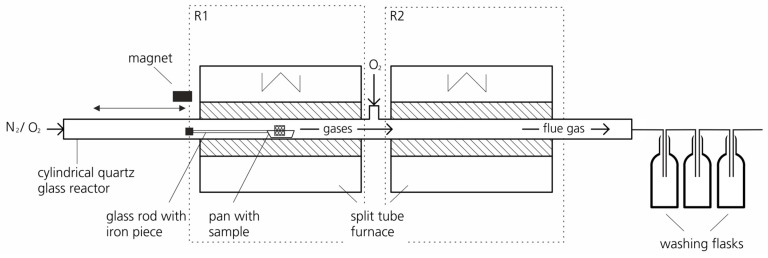
Experimental setup of a two-stage system for fluorine release analysis.

To measure fluorine release during the thermal decomposition of PV backsheets, a two-stage system with heating units surrounding a quartz glass reactor was set up (see Figure 1). 

This quartz glass reactor was chosen after considering its suitability with fluorine, as released fluorine could react with the walls or reactor lining. The length of the reactor was 1.350 m with a diameter of 0.025 m. The reactor was divided into two reaction zones, R1 and R2, where a split tube furnace (Carbolite HST 12/200) with a heating length of 0.200 m surrounded both R1 and R2. In this system, test samples could be either incinerated or pyrolyzed. The two split tube furnaces could be independently controlled and had a heating range up to 1200 °C. Reaction zone R1 served as a reaction chamber for both pyrolysis and incineration experiments. The flue gas released from the first zone (R1) of the thermal decomposition process (incineration or pyrolysis, respectively) was post-combusted in the second zone (R2) and then directed into a series of washing flasks. Here, fluorine was dissolved and could be readily analyzed using ion chromatography (IC). 

The temperature of the R1 split tube furnace was set constant during the experiments. The experimental trials were conducted for temperatures between 300 °C and 950 °C. Reaction zone R2 served as a post-combustion chamber and was therefore set to a constant temperature of 1050 °C. One end of the reactor served as an inlet while the other served as outlet. The inlet side was equipped with a borehole for purge gas entrance, and the outlet side had a similar provision for the transfer flue gases. In addition, an inlet for O_2_ supply provided oxygen flow to R2. 

At the outlet of the reactor, three washing flasks were connected to wash the flue gases. The washing flasks were filled with different alkaline absorption solutions (1 molar NaOH solution, 3.2 mmolar Na_2_CO_3_/1.0 mmolar NaHCO_3_ solution) to bind fluorine-containing gas compounds from the flue gas as fluoride. The choice of absorption solution depended on the expected fluorine concentrations to be released in the gas phase. For analyzing the absorption solution, a Metrohm IC 818 ion chromatograph with a limit of quantification (LOQ) of 0.01 mg/L was used [30]. The LOQ corresponded to 0.0125 wt. % fluorine with a net weight of 0.04 g initial sample per liter and 500 ml absorption volume. The results obtained from this were then compared with the initial sample masses to calculate fluorine transfer into the gas phase as well as the residual fluorine content that could be present in the solid residues.

The experiments were conducted under a fume hood considering the toxicity of released fluorine and other fluorinated compounds.

### 2.2. Materials and Sample Preparation

**Figure 2:**
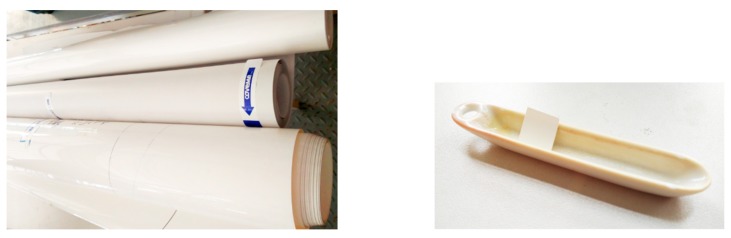
Production film (**left**); sample placed in a ceramic pan (**right**).

Three different PV backsheet materials, namely, PPE, TPT, and KPK, were obtained from COVEME S.p.A., a leading manufacturer of backsheets in 2018. The backsheets were sampled from their respective original production films (see Figure 2). Over 20 such cross sections per backsheet type were cut out from random areas across the backsheet to obtain representativeness. The sample size was 1 cm^2^ and was determined by the requirements of the laboratory-scale unit. The sample weight was found to vary between 30 and 80 mg across the film due to varying material densities and product inhomogeneity.

Prior to the experiments, all backsheet samples were oven-dried at 65 °C and analyzed for proximate analysis [31,32]. The results are shown in Table 1. The KPK sample was found to have the highest fixed carbon content and least ash content. PPE contained the highest amount of volatiles, while its ash content was similar to that of the TPT sample.

The backsheet samples were also analyzed to estimate their elemental composition for ultimate analysis according to [33,34], as well as the lower heating value according to [35]. These results are shown in Table 2.

### 2.3. Reference Experiments

Finally, ahead of the fluorine release experiments via incineration and pyrolysis pathways, reference experiments with reference materials were conducted to ensure high measurement quality and to quantify experimental or measurement errors. As a reference material, PVDF (from Sigma-Aldrich) was chosen; it contains fluorine and is one of the key materials of the backsheet type KPK. This reference material, with a high fluorine content of 59.4 wt. % (calculated by molecular weight) or 57.6 wt. % (measured via oxygen bomb combustion ion chromatography and the above described standard [30]), was incinerated at 950 °C. Furthermore, a PVDF–cellulose mixture with low fluorine content of 4.9 wt. % (calculated by molecular weight) or 5.0 wt. % (measured via oxygen bomb combustion ion chromatography and the above described standard [30]) was also incinerated at 950 °C. 

All experimental trials with backsheet materials were conducted thrice to overcome any uncertainties. The final carbon residues (resultant char) was also analyzed with respect to the residual fluorine content in the solid phase. These reference experiments were performed to test the experimental system concerning the release of fluorine. Table 5 shows the reference test results.

### 2.4. Pyrolysis Experiments

The pyrolysis experiments were conducted at 300 °C, 400 °C, 500 °C, and 900 °C, with nitrogen as the purge gas. The pyrolysis residues were weighed and incinerated at 950 °C.

Upon reaching the desired reaction temperature in the furnace of R1 (300 °C, 400 °C, 500 °C, or 900 °C) and R2, where the reactor temperature was set constant at 1050 °C, the backsheet sample placed in a ceramic pan was positioned inside the quartz glass reactor outside the furnace of R1. Attached to the pan was a quartz glass rod fixed to a piece of iron. The quartz glass reactor was then closed and purged with N_2_ at 60 L/h. After a certain time, when the furnace temperature was steady and the N_2_ purge guaranteed complete inertization, the sample was inserted slowly into reaction zone R1 using a magnet and the iron piece attached to the glass rod. The slow insertion of the sample into the heated zone was to prevent deflagration.

The sample remained in reaction zone R1 for 30 minutes, where eventual gas release occurred. Any solid residue (pyrolysis residue) was subjected to subsequent incineration experiments (refer to Section 2.5). The released gases were subsequently transported into post-combustion zone R2 by the purge gas and were combusted there at 1050 °C in the presence of O_2_ at 60 L/h. The flue gas was then passed through a set of three washing flasks. The collected content from the washing flasks was combined, filtered via a cellulose acetate filter, and analyzed using ion chromatography. 

Each test was conducted thrice and an average value was calculated. A list of experimental conditions is given in Table 3.

### 2.5. Incineration Experiments

The incineration experiments were a modified version of the pyrolysis experiments with respect to the purge gas and the reaction temperature. Here, O_2_ instead of N_2_ at a flowrate of 60 L/h was used as the purge gas. The targeted incineration temperatures were 750 °C, 850 °C, and 950 °C for reaction chamber R1. Similar to the pyrolysis experiments, the post-combustion chamber R2 was set to a constant temperature at 1050 °C. A list of experimental conditions is shown in Table 4.

## 3. Results and Discussion

### 3.1. Reference Test Experiments

The reference experiment results with the earlier-described two-stage system (see Table 5) supported the results from the standardized oxygen bomb combustion and the theoretical calculations based on the molecular weight of PVDF (reference material). The theoretical values represent the maximum fluorine content that can be released. The results show negligible differences between the theoretical content and measured values from oxygen bomb combustion or the two-stage system. Furthermore, it can also be observed that the two-stage system coped with very high concentrations, up to 60 wt. % fluorine, but was also sensitive to lower concentrations, around 5 wt. %. Therefore, the results obtained by the experimental method confirm the calculated values and deviate only slightly from the results obtained via the standardized method, thereby validating the suitability of the experimental setup.

### 3.2. Pyrolysis Experiments

**Figure 3:**
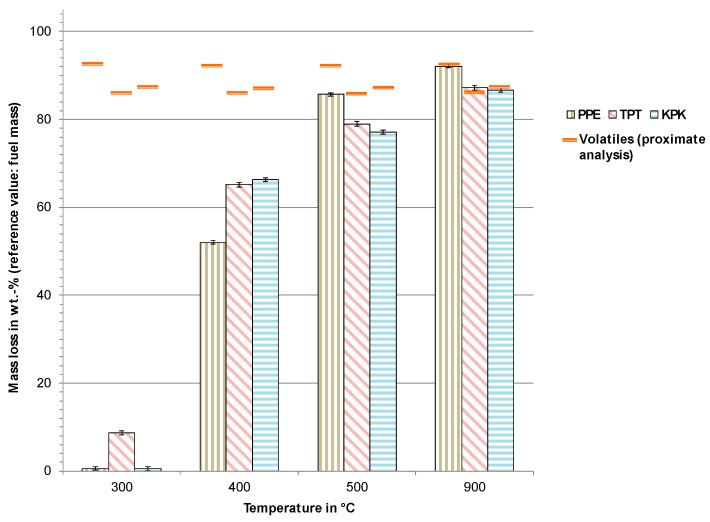
Mass loss from the pyrolysis of PPE, TPT, and KPK at various temperatures.

**Figure 4:**
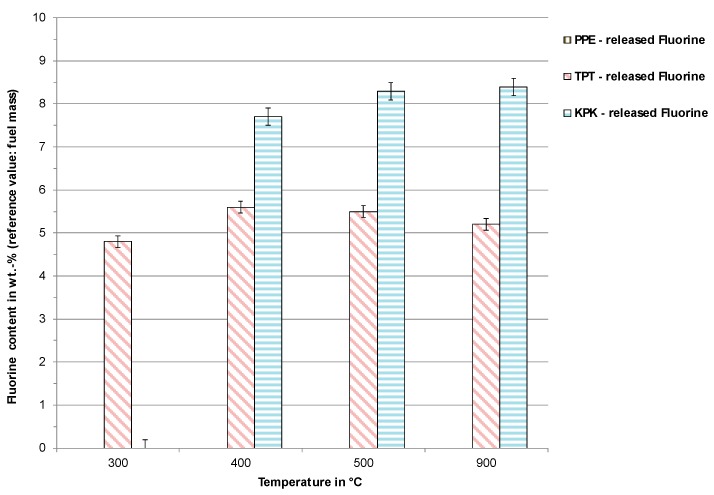
Pyrolysis results of fluorine transfer into the gas phase from PPE, TPT, and KPK.

The results from the pyrolysis experiments are shown in Figure 3 and Figure 4. Figure 3 shows the mass loss due to thermal degradation, where a slight degradation of TPT can be observed at 300 °C. For PPE and KPK, no significant mass loss was observed at this temperature. At 400 °C and 500 °C, all sample materials showed a significant mass loss. However, values comparable to the volatile content obtained by proximate analysis could only be achieved at a high temperature of 900 °C. 

The degradation behavior of Tedlar^®^-containing material corresponded to results from [36], where backsheet materials were pyrolyzed using thermogravimetric analysis (TGA). The degradation behavior of PVDF (Kynar^®^), PET, and EVA supported the studies by [37,38,39,40]. It was observed that main pyrolysis reactions occurred between 400 °C and 500 °C, and any increase in temperature beyond those values did not yield further significant mass loss. At 900 °C, a complete devolatilization was achieved and the same mass loss as obtained by proximate analysis could be measured.

Figure 4 shows the amount of fluorine released into the gas phase during pyrolysis. The values shown in wt. % refer to the original sample mass. The values demonstrate that TPT released fluorine already at 300 °C, while for KPK, this occurred at a temperature of around 400 °C. This corresponded to the mass loss measurements shown in Figure 3. KPK showed higher values of released fluorine due to higher fluorine content in the original sample. Temperatures above 400 °C only led to a relatively small increase in fluorine release compared to release at 400 °C. 

In comparison to the fluorine content from ultimate analysis (see Table 2), the data show incomplete fluorine release into the gas phase during pyrolysis. This leads us to the conclusion that the resultant pyrolysis char from these experiments could contain the remaining fluorine. 

**Figure 5:**
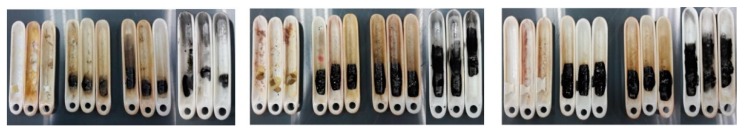
Pictures of pyrolysis char residues of each sample at 300 °C, 400 °C, 500 °C, and 900 °C for the three sample types PPE, TPT, and KPK (from **left** to **right**).

Figure 5 shows the pyrolysis char residues from the three backsheet samples at various process temperatures. The resultant residues (char) from the pyrolysis experiments were incinerated at 950 °C as described earlier. During incineration, fluorine was found to be transferred completely to the gas phase. The results found by this method confirm that the fluorine that is not transferred into the gas phase during pyrolysis ends up as compounds in the residues. 

**Figure 6:**
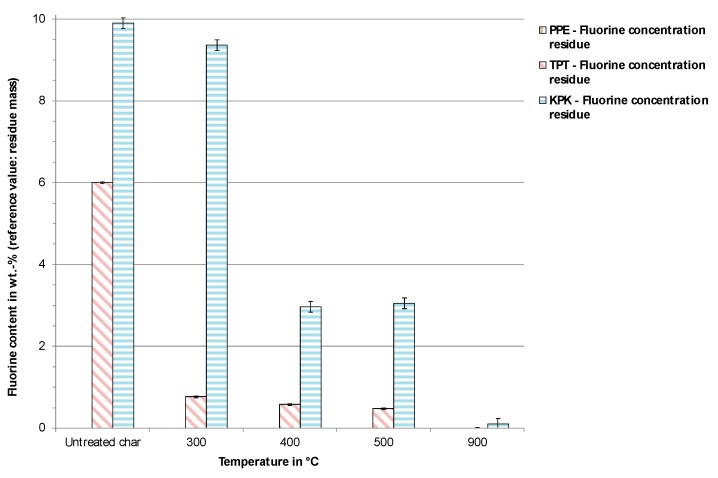
Incineration of residual pyrolysis char and fluorine concentration.

It can be observed in Figure 6 that higher amounts of fluorine from TPT were released already at lower temperatures (≤300 °C); however, to achieve complete release of fluorine, a high pyrolysis temperature (>500 °C) was needed. Similarly, in the case of KPK, the release of the majority of the fluorine compounds also occurred at temperatures higher than 300 °C, and the complete release of fluorine occurred at temperatures above 500 °C. At 900 °C, nearly all fluorine content was released into the gas phase from both backsheet materials. 

It was thus concluded that a pyrolysis temperature as high as 500 °C is required to produce fluorine-free char that could be used for energetic applications. Evidently, the char derived from the PPE did not contain any fluorine as expected. 

The possible mechanisms of PVF and PVDF degradation during pyrolysis were described in detail by [41]. We examined three mechanisms explained by [19] for the degradation of PVDF and concluded that the formation of free radicals which unzip to the monomer CH_2_=CF_2_ is a plausible mechanism. However, further studies are needed to understand and model PVF and PVDF degradation in detail. 

### 3.3. Incineration Experiments

**Figure 7:**
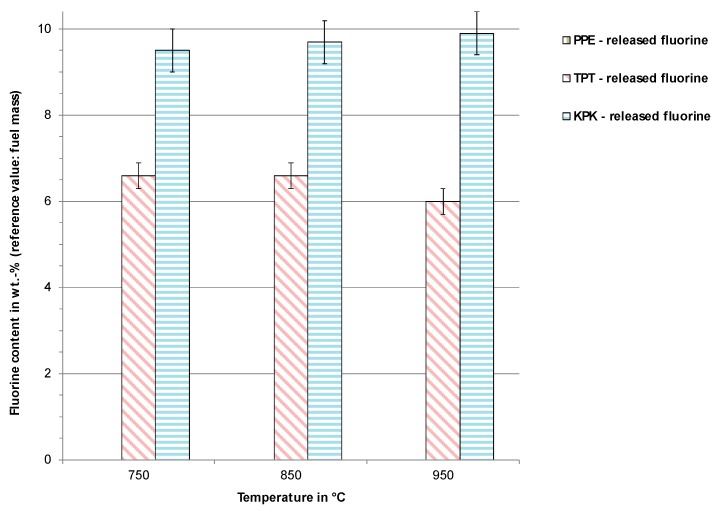
Incineration results of fluorine transfer into the gas phase from PPE, TPT, and KPK.

To examine the release behavior of fluorine compounds during incineration, the samples PPE, KPK, and TPT were incinerated at three different temperatures—750 °C, 850 °C, and 950 °C. The observed results did not vary significantly at different temperatures (see Figure 7). Indeed, the complete release of fluorine into the gas phase was observed at all three temperatures. This result was expected, as most of the volatiles are released already at temperatures lower than 750 °C. Considering the oxygen availability, ignition and exothermal reaction at much higher temperature than the oven temperatures was likely. As expected, incineration of PPE did not release any fluorine. These released fluorine values were higher than the values shown in Table 2, which is attributed to different measurement methods. 

## 4. Conclusions

Three PV backsheet materials that are commonly used in photovoltaic modules were analyzed to observe fluorine release during pyrolysis and incineration at different temperatures. Two of the materials (KPK and TPT) contained fluorine compounds, whereas the third material (PPE) was used as a control material without any fluorine compounds. It was observed that most of the fluorine was released into the gas phase during pyrolysis and incineration. Slight differences could be observed concerning the starting temperature of release during pyrolysis. A high percentage of fluorine release from TPT was observed even at temperatures lower than 300 °C. The release temperature for KPK, however, was higher (300–400 °C). Furthermore, it was observed that residues from pyrolysis at temperatures lower than 500 °C still contain fluorinated compounds. Hence, further experimental investigation and modelling is required to understand and possibly control these release phenomena, as this could be a key factor when processing fluorinated polymeric materials for energy recovery or for recycling after their EoL.

Nonetheless, from the experimental analysis presented in this study along with the literature body on the thermal degradation of fluoropolymers, it can be concluded that the EoL handling of fluorinated PV backsheets is not just challenging but also adds to the formation of other persistent compounds such as fluorocarbons, fluoroacids, furans, and dioxins. Even in hazardous waste incineration plants that are equipped with sophisticated downstream processing, studies show that significant quantities of trifluoroacetate can still be released into the environment. Hence, it can be concluded that although fluoropolymers might have some characteristic attributes such as thermal stability, they still pose risks to both human health and the environment. PPE backsheets on the other hand, are preferable from a recycling and wider the circular economy perspective.

## Figures and Tables

**Table 1:** Proximate analysis of backsheet samples.

Type of Backsheet	Fixed Carbon (wt. %)	Volatiles (wt. %)	Ash Content (wt. %)
PPE	2.9	92.2	4.9
TPT	8.2	86.1	5.7
KPK	10.7	88.2	1.1

**Table 2:** Ultimate analysis and heating values of the backsheet samples.

Type of Backsheet	C (wt. %)	H (wt. %)	O (wt. %)	N (wt. %)	F (wt. %)	Lower Heating Value (MJ/kg)
PPE	63.8	6.6	24.5	0.2	0.0	30.0
TPT	55.6	4.5	28.5	0.2	5.5	26.6
KPK	54.9	4.3	30.5	0.2	9.0	24.6

**Table 3:** Experimental conditions—pyrolysis experiments.

Material	R1 Temperature during Pyrolysis, (°C)	Purge Gas	Absorption Solution	Repetitions
PPE, TPT, KPK	300, 400, 500, 900	N_2_	Na_2_CO_3_/NaHCO_3_	3

**Table 4:** Experimental conditions—incineration experiments.

Material	R1 Temperature during Incineration, (°C)	Purge Gas	Absorption Solution	Repetitions
PPE, TPT, KPK	750, 850, 950	O_2_	Na_2_CO_3_/NaHCO_3_	3

**Table 5:** Reference test results.

Reference Material	Theoretical Fluorine Content, in wt. %	Measured Fluorine Content, Incineration at 950 °C in the two-Stage System, wt. %	Measured from Oxygen Bomb Combustion, in wt. %
PVDF	59.4	59.7	57.6
PVDF–cellulose mixture	4.9	5.1	5.0
